# Elemental composition of the hair and milk of black-spotted cows and its relationship with intestinal microbiome reorganization

**DOI:** 10.14202/vetworld.2022.2565-2574

**Published:** 2022-11-14

**Authors:** Elena Sizova, Elena Yausheva, Olga Marshinskaia, Tatiana Kazakova, Yuriy Khlopko, Svyatoslav Lebedev

**Affiliations:** 1 Federal Research Centre of Biological Systems and Agrotechnologies of the Russian Academy of Sciences, 460000 Orenburg, Russia; 2 Institute for Cellular and Intracellular Symbiosis of the Ural Branch of the Russian Academy of Sciences, 460000 Orenburg, Russia

**Keywords:** breeding, heavy metals, intestinal microbiome, probability

## Abstract

**Background and Aim:**

The cattle breeding system is facing severe problems associated with the increased negative impact of various human activity areas on the environment and the bodies of farm animals. The use of heavy metals in different production areas leads to their accumulation in the environment due to the ingestion of animals and humans through animal products. This study aimed to assess the elemental composition of the hair and milk of black-spotted cows and to identify the relationship between the content of toxic and essential elements and the state of the intestinal microbiome.

**Materials and Methods:**

The element status was estimated by studying the chemical composition of the biosubstrates using inductively coupled plasma-mass spectroscopy. Based on the analysis of hair, the elemental composition, and the use of the coefficient of toxic load, two groups of animals were formed: Group I, which included cows with a lower load factor, and Group II, which included cows with a higher load factor.

**Results:**

An increase in the heavy metal concentrations in the hair and milk of animals in Group II was observed. The As, Fe, Pb, Al, Co, Ni, and V concentrations in the hair of cows from Group II increased relative to Group I by 19%, 29%, 24.5%, 32.3%, 35.6%, 21.5%, and 18.2%, respectively. There was a significant increase in the level of Fe by 11.5%, Cr by 8.25%, Mn by 17.6%, Pb by 46.1%, and Cd by 25% in Group II compared with Group I in the assessment of elemental milk composition. There were no apparent changes in the intestinal microbiome of Group II.

**Conclusion:**

Some heavy metals were accumulated in the bodies and milk of animals. This shows a high probability of heavy metals causing harm to the health of animals and humans.

## Introduction

In recent decades, dairy farming has been gaining momentum in producing milk and dairy products worldwide [1, 2]. Dairy and other animal products play an essential role in the human diet. They are critical in children’s nutrition as good sources of protein, vitamins, sugars, and minerals [3, 4]. Milk production and quality are reduced by the deterioration of environmental conditions, affecting the level of realization of the genetic potential of animals. The cattle breeding system is currently facing severe problems associated with the increased negative impact of various areas of human activity on the environment and the bodies of farm animals [5, 6]. Heavy metals use in multiple production areas leads to their environmental accumulation, resulting in the ingestion of animals and humans through animal products [7]. The significant content of toxic chemical elements in livestock products is a highly urgent problem for both developing and developed countries [8–10]. Increased content of several heavy metals in the human body leads to disruption of the liver, kidneys, circulatory system, and central nervous system [11, 12]. In addition, the impact of heavy metals on the body of children is the cause of the development of neurodegenerative diseases [13–15]. Connected with the above, control of the physiological state, including mineral metabolism of essential and toxic elements, is an integral part of assessing the health of animals in the production of meat and dairy products.

Hair is a material frequently used to assess the elemental status of animals. The use of hair as an indicative biomaterial is associated with its informative value as a long-term parameter for determining the state of mineral metabolism [16, 17]. The microbiome of the gastrointestinal tract is one indicator of changes in the internal homeostasis of ruminants. The gut microbiome of cattle plays a fundamental role in the metabolic functions of the host and the functioning of the immune system [18, 19]. Changes in the composition of the intestinal microbiota of cattle closely correlate with indicators of productivity and the physiological state of animals [20, 21].

This study aimed to assess the elemental composition of the hair and milk of black-spotted cows. In addition, this study aimed to identify the relationship between the content of toxic and essential elements and the state of the intestinal microbiome.

## Materials and Methods

### Ethical approval

The Ethics Committee of the Federal Research Centre for Biological Systems and Agrotechnologies of the Russian Academy of Sciences approved the experimental design (No. 5 of 02/05/2021). All animal studies were performed following the ethical standards laid down in the 1964 Declaration of Helsinki and its later amendments.

### Study period and location

This study was conducted from June to August 2021. Experiments were conducted at the center of collective sharing of Biological Systems and Agrotechnologies of the Russian Academy of Sciences.

### Experimental animals

The studies were conducted on a model of black-spotted cows (n = 70; age 4–6 years; live weight 610–640 kg; and lactation stage 30–55 days after calving) in JSC “Kalinina.” (Tashlinsky District, Russia). All animals were clinically healthy. All animals were under the same conditions and pasture management. The diet of the black-spotted cows during the experiment is shown in Table-1.

**Table-1:** Ingredients and mineral compositions of the diet.

Ingredients	g of dry matter/ kg of body weight
Cereal-leguminous grass	44.5
Compound feed	11.6
Beet molasses	0.5
Table salt	0.07
Mineral composition	
Ca	184.8 g
P	110.6 g
Mg	41 g
K	178 g
Na	3514 g
Al	958.6 mg
As	1.79 mg
B	118.5 mg
Cd	1.83 mg
Co	23.9 mg
Cr	4.31 mg
Cu	290 mg
Fe	2023 mg
Hg	0.048 mg
I	27.9 mg
Li	2.05 mg
Mn	1801 mg
Ni	34.3 mg
Pb	3.81 mg
Se	2.01 mg
Si	1853 mg
Sn	0.69 mg
Sr	249 mg
V	2.15 mg
Zn	1789 mg

### Analysis of microelements

Animal hair and milk samples were used as biosubstrates to study elemental status. Hair samples weighing at least 0.4 g were taken from the upper part of the animals’ withers according to the standard technique. The proximal portion of hair was cut off 15 mm long from the root within the period closest to sampling to assess the elemental status of the organism.

The collected hair samples were washed in acetone (Khimmed, Russia) within 10–15 min. Then, the hairs were washed thrice in deionized water (18 MΩ • cm). Deionized water was obtained using an electric distiller with a combined membrane set DVS-M/1HA-1(2)-L (Mediana-Filter, Podolsk, Russia). Later, samples were dried at 60°C until they reached an air-dry state. The corresponding sample weights (about 0.05 g) were treated with 5 mL of nitric acid (Khimmed) in a Multiwave 3000 microwave system (Perkin Elmer-A. Paar, Austria) using the following mode: The temperature was raised to 200°C for 5 min and remained stable for 5 min at 200°C. Then, the temperature was cooled to 45°C. The split solutions were poured into 15 mL polypropylene tubes. Labels and caps were washed three times with deionized water. The rinsed samples were included in the appropriate tubes. Finally, the solutions were filled up to 15 mL with deionized water and thoroughly mixed by shaking closed tubes [22].

Raw milk samples were taken individually from each cow, placed in sterile containers, and cooled to 5°C. The obtained samples were diluted (1: 15; v/v) with an acidified (pH = 2.0) diluent consisting (v/v) of 1% 1-butanol (Merck KGaA, Darmstadt, Germany), 0.1% Triton X-100 (Sigma-Aldrich, Co., St. Louis, USA), and 0.07% HNO_3_ (Sigma-Aldrich) in distilled deionized water (18 MΩ cm-1) (Merck Millipore, Billerica, MA, USA) [22].

Analytical studies of the microelement composition of hair and milk according to 25 elements (e.g., Ca, Cu, Fe, Li, Mg, Mn, Ni, As, Cr, K, Na, P, Zn, I, V, Co, Se, Ti, Al, Be, Cd, Pb, Hg, Sn, and Sr) were conducted in the laboratory of the ANO Center for Biotic Medicine (Moscow). The atomic emission (Optima 2000DV, PerkinElmer Corp., USA) and mass spectral (Elan 9000”, “PerkinElmer Corp.,” USA) analyses were conducted with inductively coupled plasma.

The coefficient of the total toxic load (K_tox_) was calculated to assess the magnitude of the toxic load on the cows’ bodies. The sum of the coefficients of some heavy elements (e.g., Mn, Fe, Cu, Zn, As, Sr, Pb, Cd, and Hg) was used to calculate the coefficient of the toxic load (K_tox_):

Кtox = K_Mn_ + K_Fe_ + K_Cu_ + K_Zn_ + K_As_ + K_Sr_ + K_Pb_ + K_Cd_ + K_Hg_,

Where K_Mn_….K_Hg_ is the ratio of the content of an element in the hair of a particular cow to the content corresponding to the 50^th^ percentile.

After dividing the total number of cows into groups according to K_tox_ (Group I [n = 32] and Group II [n = 38]), five animals were selected from each group to study the intestinal microbiome. However, the apparent limitation of our study was the small number of animals included in the analysis. This occurred due to the design of the main research and the remarkably high cost. Nevertheless, these data provide new insights into the intestinal microbiome. The study of the microbiome was conducted in the large intestine (gut content) of cows. Samples were taken with a sterile instrument into a test tube with a preservative solution (DNA/RNA Shield, USA). Finally, the samples were frozen.

#### Total DNA extraction

The samples were homogenized on a TissueLyser LT (Qiagen, Hilden, Germany) with a Lysing Matrix Y (MP Biomedicals, Solon, USA). DNA extraction from samples was performed using a QIAamp Fast DNA Stool Mini Kit (Qiagen) according to the manufacturer’s instructions. The quality of the extracted DNA was assessed using electrophoresis in 1% agarose gel and a Nanodrop 8000 (Thermo Fisher Scientific, Waltham, MA, USA). The DNA concentration was quantified using a Qubit 4 Fluorometer (Life Technologies, Carlsbad, CA, USA) with a dsDNA High Sensitivity Assay Kit (Life Technologies).

#### Library preparation and sequencing

Preparation of the DNA libraries was made according to the Illumina protocol (Part #15044223, Rev. B.) with primers targeting the V3–V4 regions of the SSU ribosomal RNA (rRNA) gene, S-D-Bact-0341-b-S-17 (5′-CCTACGGGNGGCWGCAG-3′) as the forward primer and S-D-Bact-0785-a-A-21 (5′-GACTACHVGGGTATCTAATCC-3′) as the reverse primer. The reaction mixture (25 μL) contained both primers (0.2 μM of each). The mixture also contained 80 μM dNTPs and 0.2 U Q5 High-Fidelity DNA Polymerase (New England Biolabs, Ipswich, MA, USA). The following PCR program was used: 95°C for 3 min, 25 cycles: 95°C for 30 s, 56°C for 30 s, 72°C for 30 s, and final extension 72°C for 5 min. The DNA libraries were cleaned using Agencourt AMPure XP beads (Beckman Coulter, Brea, CA, USA). They were validated by capillary electrophoresis on a Qiaxcel Advanced System (Qiagen) using the QIAxcel DNA Screening Kit (Qiagen). Paired-end 2 × 251 bp sequencing was performed on the MiSeq platform (Illumina, San Diego, CA, USA) with the Reagent Kit v.2 (Illumina, San Diego, CA, USA).

DNA library preparation, sequencing, and bioinformatics treatment were performed in the Center of Shared Scientific Equipment “Persistence of Microorganisms” from the Institute for Cellular and Intracellular Symbiosis UrB RAS (Orenburg, Russia).

### Bioinformatic Processing

In the first stage, the raw reads obtained as a result of sequencing were evaluated using FastQC v. 0.11.7 (Babraham Institute, UK). The evaluation was necessary to determine the parameters of further processing and included an assessment of the quality and length of reads and the presence of adapter sequences. Paired-end reads were merged using USEARCH v10.0.240_win32 (drive5.com/usearch), using the “-fastq_mergepairs” command with the “-fastq_maxdiffs 10 -fastq_pctid 80 options.” After merging and adapter removal, the reads were re-evaluated with FastQC v. 0.11.7. Subsequent treatment of merged reads was conducted with Usearch v10.0.240_win32 (drive5.com/usearch) [1] and included quality filtering (expected error or maxee <1.00) and amplicon size selection (420 bp of minimal size). The filtering quality was evaluated with FastQC v. 0.11.7.

The next stage included dereplication and clustering of the filtered reads using the UPARSE algorithm. As a result of dereplication and clustering, operational taxonomic units (OTUs) were formed. Chimeric sequences were detected and removed using the UCHIME2 algorithm [2] during the clustering phase. Final OTUs were aligned to the initial merged reads using global alignment (usearch_global tool) at a 97% level of similarity. As a result of global alignment, the number of merged reads corresponding to every OTU was estimated. Contaminant OTUs were identified and removed through the “usearch_ublast” command by matching the sequences of the trial samples and the negative control samples. The taxonomic classification of sequences was conducted using the ribosomal database project reference database (rdp.cme.msu.edu/index.jsp) [3]. The visualization of the results of bioinformatic processing was implemented using a microbiome analyst [4]. After filtering and assigning taxonomic affiliations, the resulting OTUs were used to calculate alpha (e.g., Chao1, equitability, abundance-based coverage estimated [ACE], Fisher’s alpha, Simpson, and Shannon2 indexes [statistical method: Analysis of variance]) and beta (e.g., ordination method: non-metric multidimensional scaling; distance method: Bray-Curtis index; statistical method: Permutational Multivariate Analysis of Variance [PERMANOVA]) diversities.

### Statistical analysis

The data were processed using the methods of variation statistics and the statistical package Statistica 10 (StatSoft, USA). The differences regarding the data on the taxonomic composition of the intestinal microbiome were considered statistically significant at p ≤ 0.05 using Student’s t-test.

For data on the elemental composition of hair and milk, compliance with the normal distribution law was verified using the Kolmogorov goodness-of-fit test. The hypothesis that the data belonged to a normal distribution were rejected in all cases, with a probability of 95%. Therefore, the use of nonparametric procedures for statistically processing populations was justified, allowing the use of the Mann–Whitney U test. The data obtained are shown as median (Me) and 25–75^th^ quartiles (Q_25_–Q_75_).

## Results

According to the K_tox_ calculation, two groups were formed. Group I included cows with a lower load of K_tox_ <8.95 relative to the studied sample. Group II included cows with a higher load factor K_tox_ >8.95 relative to the study sample. Significant differences in Table-2 were revealed in the analysis of the chemical elements in the hair of dairy cows.

**Table-2:** The content of chemical elements in the hair of cows, depending on the value, К_tox_, μg/g.

Elements in hair	Group I	Group II
As	0.145 (0.125–0.155)	0.172 (0.158–0.193)*
Cd	0.008 (0.007–0.009)	0.009 (0.008–0.01)
Cu	7.73 (7.68–8.1)	7.71 (7.52–9.34)
Fe	220.0 (160.0–247.0)	284.0 (240.0–326.0)*
Hg	0.005 (0.004–0.007)	0.007 (0.005–0.008)
Mn	8.36 (7.58–9.31)	9.345 (8.07–10.88)
Sr	4.51 (3,66–5.17)	4.185 (3.79–4.6)
Zn	114.0 (110.0–117.0)	124.0 (114.0–130.0)
Pb	0.159 (0.157–0.183)	0.198 (0.185–0.215)*
Al	110.0 (104.0–124.0)	145.5 (124.0–172.0)*
B	0.808 (0.662–0.829)	0.77 (0.638–0.954)
Ca	1 318.0 (1 042.0–1 406.0)	1 188.0 (1 083.0–1 264.0)
Co	0.101 (0.089–0.117)	0.137 (0.121–0.15)*
Cr	0.664 (0.562–0.799)	0.782 (0.701–0.828)
I	0.399 (0.332–0.521)	0.409 (0.359–0.454)
K	813.0 (778.0–1 055.0)	769.5 (655.0–939.0)
Li	0.297 (0.233–0.299)	0.284 (0.257–0.307)
Mg	212.0 (166.0–258.0)	224.5 (192.0–237.0)
Na	349.0 (256.0–562.0)	262.0 (223.0–364.0)
Ni	0.386 (0.34–0.421)	0.469 (0.426–0.499)*
P	243.0 (195.0–251.0)	205.0 (177.0–233.0)
Se	0.19 (0.16–0.197)	0.183 (0.149–0.221)
Si	16.67 (15.74–23.89)	18.9 (14.76–20.04)
Sn	0.014 (0.014–0.028)	0.014 (0.012–0.021)
V	0.445 (0.404–0.471)	0.526 (0.483–0.57)*

* p ≤ 0.05-I in comparison with II

The regularity of the increase in the average values of individual element concentrations with the increase in K_tox_ was revealed by the content evaluation of chemical elements in cows’ hair. The As, Fe, Pb, Al, Co, Ni, and V concentrations in the hair of cows from Group II have significantly increased relative to Group I by 19%, 29%, 24.5%, 32.3%, 35.6%, 21.5%, and 18.2%, respectively. In addition, an increase in the level of several other elements (e.g., Hg, Mn, Zn, Si, and Mg) was observed in experimental Group II in comparison with group I. A decrease in the concentration of the elements Ca, K, Na, P, Se, B, and Sr was the exception when Groups I and II were compared.

**Figure-1:**
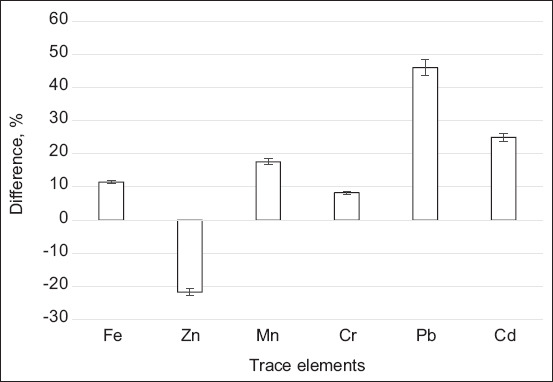
Significant changes (p ≤ 0.05) in the content of a number of chemical elements in the milk of Group II relative to the indicators of Group I.

There were no significant differences between the experimental groups for most of the studied elements of the composition of the cows’ milk, according to the analysis of the elements (Table-3). At the same time, significant changes in values were observed for several chemical elements contained in milk. There were significant increases in the levels of Fe (11.5%), Cr (8.25%), Mn (17.6%), Pb (46.1%), and Cd by 25% in Group II compared with Group I. However, there was a 21.8% decrease in Zn levels (Figure-1).

**Table-3:** Content of chemical elements in milk of cows from Groups I and II, μg/g.

Element	Group I	Group II
Al	0.09 (0.07–0.12)	0.097 (0.07–0.11)
As	0.001 (0.0008–0.002)	0.001 (0.0008–0.002)
B	0.19 (0.18–0.21)	0.184 (0.16–0.2)
Ca	1053 (1000–1120)	982.5 (896–1114)
Co	0.002 (0.0018–0.0022)	0.0021 (0.0018–0.0024)
Hg	0.00018 (0.00016–0.00048)	0.00018 (0.00016–0.00047)
I	0.0057 (0.0044–0.009)	0.0052 (0.0043–0.0087)
K	1576 (1522–1686)	1640 (1533–1701)
Li	0.03 (0.02–0.04)	0.034 (0.02–0.044)
Mg	113.5 (99.3–120.5)	111 (96.7–118.2)
Na	460.5 (419.7–510.5)	478 (431–524.3)
Ni	0.04 (0.03–0.05)	0.04 (0.03–0.06)
P	1080 (1006–1110)	1124 (1020–1173)
Se	0.027 (0.025–0.031)	0.026 (0.025–0.03)
Si	1.93 (1.82–2.26)	1.89 (1.81–2.2)
Sn	0.00068 (0.00011–0.0037)	0.00068 (0.00011–0.0036)
Sr	0.86 (0.7–1.01)	0.88 (0.71–1.01)
V	0.0096 (0.0082–0.01)	0.0096 (0.0081–0.01)
Cu	0.028 (0.025–0.0315)	0.0285 (0.0265–0.0335)

There were significant Spearman correlations between changes in the content of several chemical elements in hair and those in milk (Table-4). There were strong negative correlations between Al concentration in hair and Cr in milk (r = −0.86) and Mn in hair and Fe in milk (r = −0.94). Moderate negative correlations were observed for As in hair and Fe in milk (r = −0.54), Pb in hair and Zn in milk (r = −0.67), and Fe in air and Cr in milk (r = −0.68). Finally, there were moderate positive correlations for Pb in hair and Cd in milk (r = 0.61), Al in hair and Pb in milk (r = 0.67), Fe in hair and Fe in milk (r = 0.57), Cd in hair and Cd in milk (r = 0.58), and Mn in hair and Mn in milk (r = 0.56).

**Table-4:** Spearman’s correlation coefficient between the content of heavy metals in hair and milk of cows of Group II.

Milk elements	Hair elements						

	Al	As	Pb	Cd	Mn	Fe	Zn
Fe	−0.29	−0.54*	0.18	−0.21	−0.94*	0.57*	−0.06
Zn	−0.34	0.08	−0.67*	0.27	0.32	0.05	0.15
Mn	−0.44	−0.38	−0.18	−0.34	0.56*	−0.33	0.2
Cr	−0.86*	−0.39	−0.33	0.12	−0.27	−0.68*	0.13
Pb	0.67*	0.40	0.27	−0.41	0.24	0.4	−0.1
Cd	−0.25	−0.36	0.61*	0.58*	−0.34	−0.45	−0.27

* The correlation is significant at the level of p ≤ 0.05

There was a high diversity of taxonomic groups in the analysis of the intestinal microbiota of cows. As a result of sequencing, 301,938 reads were obtained, from 26,613 to 33,808 initial reads per sample. After the merging and filtering steps, 253,319 reads were included in the analysis. After clustering, a total of 516 OTUs were obtained. After removing singletons and doublets from the samples, 496 OTUs remained. It should be noted that 21 OTUs were found in only one of the ten samples. Therefore, they were removed from further analysis.

**Figure-2:**
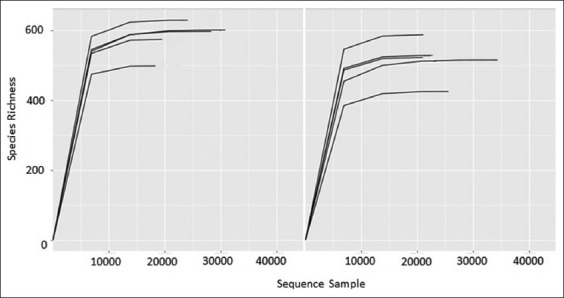
Sequence-based resolution curves for gut microbiota samples from Groups I (right) and II (left).

Based on the obtained sequences and OTUs, the resolution curves were plotted. The resolution curves of all samples tended to plateau to a maximum, indicating the sufficiency of the sequencing depth for characterizing ruminal microbiota in this study (Figure-2). The resulting OTUs were taxonomically grouped from the phylum to the genus levels and assigned to 12 phyla, 22 classes, 27 orders, 43 families, and 92 genera in Group I and 11 phyla, 21 classes, 26 orders, 42 families, and 91 genera in Group II.

**Figure-3:**
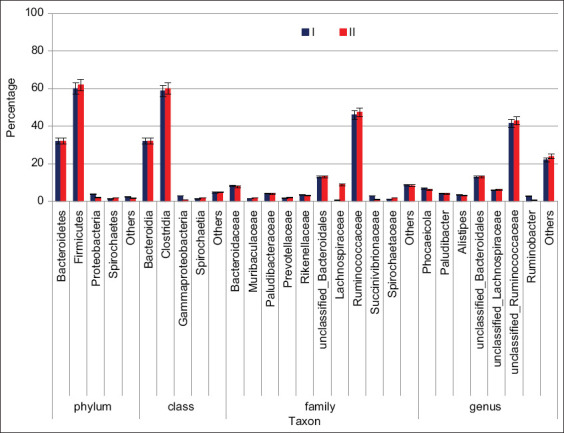
Comparative chart of the microbial community.

Firmicutes (32.1%) and Bacteroidetes (60.1%) were the most numerous phyla in samples from cows in Group I. Proteobacteria (3.98%) and Spirochaetes (1.37%) were among the least frequent phyla (Table-5). At deeper taxonomic levels in the samples of the intestinal microbiota of cows, numerous taxa were the following classes: Bacteroidia (32.1%) and Clostridia (58.8%), families *Ruminococcaceae* (46.1%), unclassified *Bacteroidales* (13%), *Lachnospiraceae* (8, 72%), and *Bacteroidaceae* (8.35%). The most abundant classified microorganisms at the genus level belonged to unclassified *Ruminococcaceae* (41.6%) and unclassified *Bacteroidales* (13%). A similar ratio of the main taxonomic groups of microorganisms of the intestinal microbiota was observed in samples of cows in Group II. There was a significant decrease in the number of microorganisms belonging to the class *Gammaproteobacteria* (*Ruminobacter*) by 2% (p = 8.82) (Figure-3).

**Table-5:** Taxonomic diversity of intestinal microbiota of the black-spotted cows, %.

Group	Taxon			

	Phylum	Class	Family	Genus
I	*Firmicutes* (60.1 ± 1.01)	*Clostridia* (58.8 ± 1.13)	*Lachnospiraceae* (8.72 ± 0.77) *Ruminococcaceae* (46.1 ± 0.93)	*unclassified_Lachnospiraceae* (5.99 ± 0.62) *unclassified_Ruminococcaceae* (41.6 ± 0.95)
	*Proteobacteria* (3.98 ± 0.41)	*Gammaproteobacteria* (3.0 ± 0.23)	*Succinivibrionaceae* (3.0 ± 0.23)	*Ruminobacter* (2.74 ± 0.27)
	*Bacteroidetes* (32.1 ± 1.21)	*Bacteroidia* (32.1 ± 1.21)	*Bacteroidaceae* (8.35 ± 0.42)	*Phocaeicola* (6.9 ± 0.39)
			*Muribaculaceae* (1.54 ± 0.83)	-
			*Paludibacteraceae* (4.26 ± 0.55)	*Paludibacter* (4.26 ± 0.55)
			*Prevotellaceae* (1.71 ± 0.53)	-
			*Rikenellaceae* (3.53 ± 0.16)	*Alistipes* (3.34 ± 0.15)
			*unclassified_Bacteroidales* (13.0 ± 1.27)	*unclassified_Bacteroidales* (13.0 ± 1.27)
	*Spirochaetes* (1.37 ± 0.43)	*Spirochaetia* (1.37 ± 0.43)	*Spirochaetaceae* (1.23 ± 0.42)	-
	Others (2.45 ± 0.24)	Others (4.73 ± 0.45)	Others (8.56 ± 0.86)	Others (22.2 ± 1.03)
II	*Firmicutes* (61.9 ± 0.89)	*Clostridia* (60.1 ± 1.41)	*Lachnospiraceae* (8.81 ± 1.68)	*unclassified_Lachnospiraceae* (6.1 ± 1.24)
			*Ruminococcaceae* (47.5 ± 1.95)	*unclassified_Ruminococcaceae* (42.9 ± 1.92)
	*Proteobacteria* (2.2 ± 1.02)	*Gammaproteobacteria* (0.88 ± 0.07*)	*Succinivibrionaceae* (1.0 ± 0.19*)	*Ruminobacter* (0.7 ± 0.06*)
	*Bacteroidetes* (32.2 ± 1.0)	*Bacteroidia* (32.2 ± 1.0)	*Bacteroidaceae* (7.81 ± 0.33)	Phocaeicola (6.12 ± 0.28)
			*Muribaculaceae* (2.0 ± 0.09)	-
			*Paludibacteraceae* (4.07 ± 0.42)	*Paludibacter* **(**4.07 ± 0.42)
			*Prevotellaceae* (2.24 ± 0.44)	-
			*Rikenellaceae* (3.11 ± 0.33)	*Alistipes* (3.01 ± 0.32)
			*unclassified_Bacteroidales* (13.1 ± 1.29)	*unclassified_Bacteroidales* (13.1 ± 1.29)
	*Spirochaetes* (1.96 ± 0.34)	*Spirochaetia (*1.96 ± 0.34)	*Spirochaetaceae* (1.95 ± 0.36)	-
	Others (1.74 ± 0.43)	Others (4.86 ± 0.39)	Others (8.41 ± 0.62)	Others (24.0 ± 0.83)

* Differences between groups are significant at p ≤ 0.05 (according to Student’s t-test)

**Figure-4:**
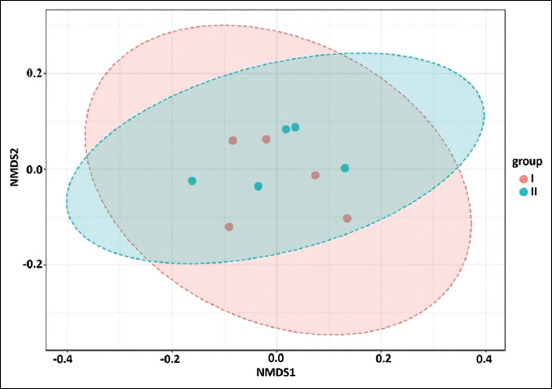
Beta diversity of intestinal microbiotas in cows of Groups I and II using Permutational Multivariate Analysis of Variance statistical method, non-metric multivariate scaling and Bray-Curtis dissimilarity.

Considering the alpha diversity calculations, it was possible to assess the richness, diversity, and homogeneity of the intestinal microbiota of cows in experimental Groups I and II (Table-6). The values of the Chao1, ACE, and Simpson indices indicated the taxonomic richness of the intestinal microbiota of the experimental groups and the absence of a predominance of one large OTU in the samples. At the same time, no significant differences between the experimental groups were observed according to Chao1, ACE, and Simpson indices. Similarly, no significant differences between the experimental groups were indicated by the diversity indices (i.e., Shannon2 and Fisher’s alpha). There was no significant effect of the level of heavy metal loading on the Bray-Curtis distance according to the PERMANOVA analysis for assessing beta diversity (Figure-4). There were no significant differences in the organization of intestinal bacterial communities between samples from Groups I and II (p > 0.05).

**Table 6:** Alpha-diversity of the intestinal microbiota of cows of Groups I and II.

Indicator	Group I	Group II	p-value
chao1	356.4	362.6	0.75
ACE	358.8	362.4	0.75
Fisher's alpha	67.425	67.44	0.73
simpson	0.04	0.04	0.67
shannon	6.65	6.63	0.98

## Discussion

The intake of heavy metals in the bodies of farm animals is one of the most critical issues in modern agricultural production. Heavy metal accumulation in soil caused by human economic activities leads to the inevitable entry of these elements into the bodies of farm animals. Therefore, it is necessary to assess the condition of animals and food products of animal origin [23, 24].

The content analysis of chemical elements in the hair of cows made it possible to divide the experimental animals into two groups characterized by high and low K_tox_ values. Discrepancies in K_tox_ values are probably more due to differences in the level of metabolic processes.

A comparison of the content of chemical elements in the hair of cows from Groups I and II (Q_25_–Q_75_ percentile intervals) was recommended to obtain the range of optimal values of the content of chemical elements. It was shown that there were similar deviations for the animals in both groups in this analysis [25]. Higher contents of Na, K, Mg, I, and Se, and lower levels of Co, As, and Cr in the hair of animals were noted in Groups I and II. However, for animals from the group with high K_tox_, a more pronounced deviation in the content of chemical elements in hair from the recommended physiological range was observed compared to animals from the group with low K_tox_. Nevertheless, such discrepancies in the content of individual elements in the hair of cows with the recommended physiological standard were probably due to more differences in the geographical zones of animal habitation, the level of metabolic processes, age, and the genotype of animals [26–28].

It was shown in the comparison of the experimental data obtained from both groups that a higher content of heavy metals in the hair of animals is accompanied by a change in the metabolism of other chemical elements in cows’ hair. Indeed, the elemental hair profile of dairy cows from Group II was characterized by increased heavy metals. Thus, as K_tox_ increased, many of the assessed macro-and microelements in the hair of individuals from Group II exceeded the same level in individuals from Group I or were significantly reduced. Previously, similar results were obtained for Holstein cows. Against the background of an increase in Pb levels in hair, higher concentrations of Co, Cr, Fe, Mn, and I were registered [29]. The trend toward high levels of Cd, Hg, and Zn in animal hair was accompanied by a high concentration of Co and I in hair, and reduced content of Ca, K, P, and Se [30]. Similar results were obtained for the racehorse model. As the content of toxic metals in the hair of horses’ mane increased, there was a significant increase in Co, Fe, Mn, V, and As concentrations and a decrease in B and I concentrations [31].

Such changes in the level of chemical elements in the body of animals are of particular interest, given research data on their correlation with changes in the productivity of animals and the quality of milk. An increase in the content of several heavy metals in hair correlates with a decrease in cows’ milk production, according to previous studies [30]. An increased intake of heavy metals into the body of cattle was registered in several studies to be correlated with a change in the elemental composition of milk and with a decrease in quality indicators of dairy products, such as protein, percentage of fat, and lactose of milk [32].

However, deviations in the metabolism of several elements in the body of cows of Group II were insufficient for a pronounced change in the composition of their intestinal microbiome. It was noted that there were no differences in the taxonomic richness of the intestinal microbiomes of cows in Groups I and II. Perhaps the absence of apparent changes in the intestinal microbiome against the background of an increase in the concentration of heavy metals is due to the high resistance of specific bacterial strains and their ability to detoxify them [33].

Among the significant changes in the intestinal microbiome of Group II cows, there was a tendency to increase the number of bacteria belonging to the class Gammaproteobacteria (*Ruminobacter*). The prerequisites for an increase in the number of *Ruminobacter* species, which destroy polysaccharides in the gastrointestinal tract of cattle, are probably due to the ability of some heavy metals to reduce the activity of gastric enzymes, such as alpha-amylase [34].

A likely consequence of changes in the elemental composition of hair, indicating the presence of deviations in the metabolism of several chemical elements in the body of cows, was a change in the level of some elements in milk. An increase in Fe, Mn, Pb, and Cd contents in milk was registered. The percentage of increase in Mn, Pb, and Cd in the milk of animals from Group II was significantly higher than in hair by 5.8%, 21.6%, and 12.5%, respectively, which was probably due to the cumulative effect. The exception was Fe, which accumulated 17.5% less in milk than in the cows’ hair. We have noted a correlation (moderate and strongly pronounced) between changes in toxic metal concentrations in hair and milk. A similar pattern was observed in other studies. An increase in the content of Cd and Pb in the blood and urine of animals is significantly correlated with an increase amounting to these metals in milk [35, 36].

The observed increase in the concentration of several toxic elements in the hair and milk of animals from Group II requires clarification about the probable sources of heavy metals and the reasons for their accumulation in the bodies of the analyzed animals. As part of the study, there was no artificial introduction of supplements with a high concentration of several heavy metals in the diet. In this regard, the most probable sources for the production of heavy metals were water and fodder obtained under free-range conditions (pasture maintenance). A significant correlation between the levels of some elements (e.g., As, Pb, and Cd) in animal feed, water, and milk has been noticed in previous studies [32]. Despite their low concentrations in diet and water, the increase in the content of toxic metals in the bodies of animals is probably a cumulative effect, which is noted for some of them. In the literature, we have found that low Cd and Pb consumption in feed does not lead to their sharp accumulation in dairy products but leads to their accumulation and preservation for a long time in the kidneys, liver, and bones. At the same time, the increased concentration of some heavy metals in milk and the body has been described [37].

The probable reason for the accumulation of certain metals in the body of some cows under the same conditions of keeping may be differences in the level of metabolic processes in the body. For example, it was noted in previous studies that elements such as Cu, Mn, and Zn accumulate in higher concentrations in multiparous cows compared to primiparous cows [38]. The interrelation of the concentration of some heavy metals in the hair of cows with their productivity was noted. For example, low productivity indicators correlate with increased concentrations of lead in the hair of cows [39]. Therefore, it is likely that fluctuations in the pool of elements in the body of animals indirectly depend on factors affecting productivity. Among these factors, oxidative stress can be indicated, the development of which is closely related to milk yield. Highly productive cows develop oxidative stress at the lactation peak, which is reflected in an increase in the malondialdehyde level in milk and a decrease in nutritional value. After lactation peak, metabolic status stabilizes, and malondialdehyde levels decrease. Cows producing substantial amounts of milk are more likely to be exposed to oxidative stress, affecting milk yield, quality, and all bodily metabolic processes [40].

At the same time, the metabolic activity of the rumen and intestine microbiomes of cows is also one of the factors of differences in metabolic processes in their bodies. The metabolic outcome of toxic substances from the environment may be altered by the microbiome of the gastrointestinal tract, altering the absorption rate of these substances [41]. In addition, changes in the metabolic pool of toxic elements may be due to the efficiency of the cows’ heavy metal detoxification system, which is represented by metallothionein proteins [42, 43].

## Conclusion

We observed an increase in the concentration of heavy metals in the hair and milk of animals in Group II. However, we did not observe significant changes in the concentrations of most of the essential elements (e.g., Ca, P, Na, Co, K, and Se) in animal milk. In contrast, their content in animal hair was reduced. We also did not observe reorganization signs of the intestinal microbiome in animals with an increased coefficient of toxic load. Changes in the metabolism of elements in the organism of animals in Group II were not critical. A more extended period of exposure would probably be required for the appearance of apparent shifts in the mineral composition of milk. At the same time, a high probability of a negative impact on the health of animals and humans may be shown by the tendency to accumulate heavy metals in hair and milk. In this regard, it is necessary to monitor the state of animals further and dynamically observe a broader range of animal health indicators. Such indicators include analysis of the activity of metal-specific proteins in the blood of animals, metabolic pathways of the intestinal microbiome, and the relationship between levels of oxidative stress and changes in the metabolic pool of micro and macronutrients.

### Data availability

Authors can confirm that all relevant data are included in the article and/or its supplementary information files. The authors declare that the data supporting the findings of this study are available within the article Miroshnikov SA, Skalny AV, Zavyalov OA, Frolov AN, Grabeklis AR (2020) The Reference Values of Hair Content of Trace Elements in Dairy Cows of Holstein Breed. Biol Trace Elem Res Mar 194:145-151. https://doi.org/10.1007/s12011-019-01768-6.

## Authors’ Contributions

All authors contributed to the study’s conception and design. TK and OM: Material preparation, data collection, and analysis. EY, YK, ES, and SL: Drafted the manuscript. All authors revised the manuscript and approved the final manuscript.

## References

[1] Gross J.J, Bruckmaier R.M (2019). Invited review:Metabolic challenges and adaptation during different functional stages of the mammary gland in dairy cows:Perspectives for sustainable milk production. J. Dairy Sci.

[2] Golder H.M, Rossow H.A, Lean I.J (2019). Effects of in-feed enzymes on milk production and components, reproduction, and health in dairy cows. J. Dairy Sci.

[3] Roman S, Sanchez-Siles L.M, Siegrist M (2017). The importance of food naturalness for consumers:Results of a systematic review. Trends Food Sci. Technol.

[4] Iannotti L.L (2018). The benefits of animal products for child nutrition in developing countries. Rev. Sci. Tech.

[5] Ni J.Q, Erasmus M.A, Croney C.C, Li C, Li Y (2021). A critical review of advancement in scientific research on food animal welfare-related air pollution. J. Hazard. Mater.

[6] Idris A.M, Said T.O, Brima E.I, Sahlabji T, Alghamdi M.M, El-Zahhar A.A (2019). Assessment of contents of selected heavy metals in street dust from Khamees-Mushait city, Saudi Arabia using multivariate statistical analysis, GIS mapping, geochemical indices and health risk. Fresen. Environ. Bull.

[7] Rai P.K, Lee S.S, Zhang M, Tsang Y.F, Kim K (2019). Heavy metals in food crops:Health risks, fate, mechanisms, and management. Environ. Int.

[8] Miclean M, Cadar O, Levei E.A, Roman R, Ozunu A, Levei L (2019). Metal (Pb, Cu, Cd, and Zn) transfer along food chain and health risk assessment through raw milk consumption from free-range cows. Int. J. Environ. Res. Public Health.

[9] Bilandžić N, Sedak M, Čalopek B, Luburić Đ.B, Solomun Kolanović B, Varenina I, Đokić M, Kmetič I, Murati T (2016). Lead concentrations in raw cow and goat milk collected in rural areas of Croatia from 2010 to 2014. Bull. Environ. Contam. Toxicol.

[10] Sobhanardakani S (2018). Human health risk assessment of Cd, Cu, Pb and Zn through consumption of raw and pasteurized cow's milk. Iran J. Public Health.

[11] Genchi G, Sinicropi M.S, Lauria G, Carocci A, Catalano A (2020). The effects of cadmium toxicity. Int. J. Environ. Res. Public Health.

[12] Nkwunonwo U.C, Odika P.O, Onyia N.I (2020). A review of the health implications of heavy metals in food chain in Nigeria. Sci. World J.

[13] Kim W, Jang Y, Lim Y.H, Kim B.N, Shin C.H, Lee Y.A, Kim J.I, Hong Y.C (2020). The effect of prenatal cadmium exposure on attention-deficit/hyperactivity disorder in 6-year-old children in Korea. J. Prev. Med. Public Health.

[14] Khan M.R, Ahmad N, Ouladsmane M, Azam M (2021). Heavy metals in acrylic color paints intended for the school children use:A potential threat to the children of early age. Molecules.

[15] Goel A, Aschner M (2021). The effect of lead exposure on autism development. Int. J. Mol. Sci.

[16] Pieper L, Wall K, Müller E, Roder A, Staufenbiel R (2016). Evaluation of sulfur status in dairy cows in Germany. Tierarztl Prax Ausg G Grosstiere Nutztiere.

[17] Zhao X.J, Li Z.P, Wang J.H, Xing X.M, Wang Z.Y, Wang L, Wang Z.H (2015). Effects of chelated Zn/Cu/Mn on redox status, immune responses and hoof health in lactating Holstein cows. J. Vet. Sci.

[18] Alipour M.J, Jalanka J, Pessa-Morikawa T, Kokkonen T, Satokari R, Hynönen U, Iivanainen A, Niku M (2018). The composition of the perinatal intestinal microbiota in cattle. Sci. Rep.

[19] Muñoz-Vargas L, Opiyo S.O, Digianantonio R, Williams M.L, Wijeratne A, Habing G (2018). Fecal microbiome of periparturient dairy cattle and associations with the onset of Salmonella shedding. PLoS One.

[20] Myer P.R (2019). Bovine genome-microbiome interactions:metagenomic frontier for the selection of efficient productivity in cattle systems. mSystems.

[21] Kim M, Park T, Yu Z (2017). Metagenomic investigation of gastrointestinal microbiome in cattle. Asian Australas J Anim.

[22] Miroshnikov S, Kharlamov A, Zavyalov O, Frolov A, Duskaev G, Bolodurina I, Arapova O (2015). Method of sampling beef cattle hair for assessment of elemental profile. Pak. J. Nutr.

[23] Licata P, Trombetta D, Cristani M, Giofrе F, Martino D, Calо M, Naccari F (2004). Levels of “toxic”and “essential”metals in samples of bovine milk from various dairy farms in Calabria, Italy. Environ. Int.

[24] Jie C, Jingzhang C, Manzhi T, Zitong G (2002). Soil degradation:A global problem endangering sustainable development. J. Geogr. Sci.

[25] Miroshnikov S.A, Skalny A.V, Zavyalov O.A, Frolov A.N, Grabeklis A.R (2020). The reference values of hair content of trace elements in dairy cows of Holstein breed. Biol. Trace Elem. Res.

[26] Zwierzchowski G, Ametaj B.N (2019). Mineral elements in the raw milk of several dairy farms in the province of Alberta. Foods.

[27] Król J, Wawryniuk A, Brodziak A, Barłowska J, Kuczyńska B (2020). The effect of selected factors on the content of fat-soluble vitamins and macro-elements in raw milk from Holstein-Friesian and Simmental cows and acid curd cheese (Tvarog). Animals (Basel).

[28] Denholm S.J, Sneddon A.A, McNeilly T.N, Bashir S, Mitchell M.C, Wall E (2019). Phenotypic and genetic analysis of milk and serum element concentrations in dairy cows. J. Dairy Sci.

[29] Miroshnikov S.A, Zavyalov O.A, Frolov A.N (2019). Influence of lead concentration in hair on interelemental interaction and milk productivity of Holstein cows. Anim. Husbandry Fodder Prod.

[30] Miroshnikov S, Notova S, Kazakova T, Marshinskaia O (2021). The total accumulation of heavy metals in body in connection with the dairy productivity of cows. Environ. Sci. Pollut. Res.

[31] Kalashnikov V, Zaitsev A, Atroschenko M, Miroshnikov S, Frolov A, Zavyalov O (2019). The total content of toxic elements in horsehair given the level of essential elements. Environ. Sci. Pollut Res. Int.

[32] Zhou X, Zheng N, Su C, Wang J, Soyeurt H (2019). Relationships between Pb, As, Cr, and Cd in individual cows'milk and milk composition and heavy metal contents in water, silage, and soil. Environ. Pollut.

[33] Neethu C.S, Mujeeb Rahiman K.M, Saramma A.V, Mohamed Hatha A.A (2015). Heavy-metal resistance in Gram-negative bacteria isolated from Kongsfjord, Arctic. Can. J. Microbiol.

[34] Kišidayová S, Pristaš P, Zimovčáková M, Blanár Wencelová M, Homol'ová L, Mihaliková K, Čobanová K, Grešáková Ľ, Váradyová Z (2018). The effects of high dose of two manganese supplements (organic and inorganic) on the rumen microbial ecosystem. PLoS One.

[35] Chirinos-Peinado D.M, Castro-Bedriñana J.I (2020). Lead and cadmium blood levels and transfer to milk in cattle reared in a mining area. Heliyon.

[36] Numa Pompilio C.G, Francisco C.S, Marco Tulio F.M, Sergio Samuel S.M, Fernanda Eliza G.J (2021). Heavy metals in blood, milk and cow's urine reared in irrigated areas with wastewater. Heliyon.

[37] Sharma R.P, Street J.C, Shupe J.L, Bourcier D.R (1982). Accumulation and depletion of cadmium and lead in tissues and milk of lactating cows fed small amounts of these metals. J. Dairy Sci.

[38] Nemec L.M, Richards J.D, Atwell C.A, Diaz D.E, Zanton G.I, Gressley T.F (2012). Immune responses in lactating Holstein cows supplemented with Cu, Mn, and Zn as sulfates or methionine hydroxy analogue chelates. J. Dairy Sci.

[39] Miroshnikov S, Zavyalov O, Frolov A, Sleptsov I, Sirazetdinov F, Poberukhin M (2019). The content of toxic elements in hair of dairy cows as an indicator of productivity and elemental status of animals. Environ. Sci. Pollut. Res. Int.

[40] Kapusta A, Kuczyńska B, Puppel K (2018). Relationship between the degree of antioxidant protection and the level of malondialdehyde in high-performance Polish Holstein-Friesian cows in peak of lactation. PLoS One.

[41] Collins S.L, Patterson A.D (2020). The gut microbiome:An orchestrator of xenobiotic metabolism. Acta Pharm. Sin. B.

[42] Kimura T, Kambe T (2016). The Functions of metallothionein and ZIP and ZnT transporters:An overview and perspective. Int. J. Mol. Sci.

[43] Juárez-Rebollar D, Rios C, Nava-Ruíz C, Méndez-Armenta M (2017). Metallothionein in brain disorders. Oxid. Med. Cell. Longev.

